# Affordable IgY-based antiviral prophylaxis for resource-limited settings to address epidemic and pandemic risks

**DOI:** 10.7189/jogh.12.05009

**Published:** 2022-02-26

**Authors:** Carrie J Chen, Anna F Hudson, Allison S Jia, Caitlin R Kunchur, Andrew J Song, Edward Tran, Chris J Fisher, Davide Zanchi, Lucia Lee, Stephen Kargotich, Mary Romeo, Ana Koperniku, Ravinder D Pamnani, Daria Mochly-Rosen

**Affiliations:** 1 Office of the Vice Provost for Undergraduate Education, Stanford University, Stanford, California, USA; 2 Department of Comparative Medicine, Stanford University School of Medicine, Stanford, California, USA; 3 Graduate School of Business, Stanford University, Stanford, California, USA; 4 Department of Chemical and Systems Biology, Stanford University School of Medicine, Stanford, California, USA; 5 SPARK at Stanford, Stanford University School of Medicine, Stanford, California, USA; 6 Stanford Byers Center for Biodesign, Stanford University, Stanford, California, USA

## Abstract

**Background:**

The COVID-19 pandemic caused by SARS-CoV-2 exposed a global problem, as highly effective vaccines are challenging to produce and distribute, particularly in regions with limited resources and funding. As an alternative, immunoglobulins produced in eggs of immunized hens (IgY) can be a simple and inexpensive source for a topical and temporary prophylaxis. Here, we developed a method to extract and purify IgY antibodies from egg yolks of hens immunized against viral pathogen-derived proteins using low-cost, readily available materials, for use in resource-limited settings.

**Methods:**

Existing protocols for IgY purification and equipment were modified, including extraction from yolks and separation of water-soluble IgY using common household reagents and tools. A replacement for a commercial centrifuge was developed, using a home food processor equipped with a 3D printed adapter to enable IgY precipitation. IgY purification was verified using standard gel electrophoresis and Western blot analyses.

**Results:**

We developed a step-by-step protocol for IgY purification for two settings in low- and middle-income countries (LMIC): a local laboratory, where commercial centrifuges are available, or a more rural setting, where an alternative for expensive centrifuges can be used. Gel electrophoresis and Western blot analyses confirmed that the method produced highly enriched IgY preparation; each commercial egg produced ~ 90 mg of IgY. We also designed a kit for IgY production in these two settings and provided a cost estimate of the kit.

**Conclusion:**

IgY purified from eggs of immunized local hens can offer a fast and affordable prophylaxis, provided that purification can be performed in a resource-limited setting. Here, we created a low-cost method that can be used anywhere where electricity is available using inexpensive, readily available materials in place of costly, specialized laboratory equipment and chemicals. This procedure can readily be used now to make an anti-SARS-CoV-2 prophylaxis in areas where vaccines are unavailable, and can be modified to combat future threats from viral epidemics and pandemics.

With over five million deaths, the rapid spread of severe acute respiratory syndrome coronavirus 2 (SARS-CoV-2) created a major global health crisis [1], which experts expect is unlikely to be the last crisis of this type [2]. The development of a diverse array of rapidly deployable and scalable solutions will be critical for ongoing protection of the population from future pandemics.

Although highly effective vaccines against SARS-CoV-2 were developed in record time [3], a number of challenges arose. Vaccine distribution has been inefficient, and even now, a year after their approval, vaccines are more accessible to higher-income countries; only 5% of people in low-income countries have received at least one dose of any of the anti-SARS-CoV-2 vaccines [4]. Availability of vaccines in resource-poor regions is limited due to the need for costly infrastructure and specialized equipment required for production, distribution, and storage. Very cold chain conditions are required for vaccine transportation and storage. The challenges in responding effectively to COVID-19 indicate that there remains a need to develop simple, low-cost protective approaches that can be produced quickly, regardless of access to expensive laboratory equipment.

Polyclonal antibodies that are produced in food animals and then purified have been extensively used to provide transient protection from infective agents in the form of passive vaccines, especially in farmed animals [5]. Immunoglobulin (IgY) produced by hens has shown promise when used against bacterial and viral infections [6]. Antibodies produced by immunizing hens with a protein from the virus offer a practical passive vaccination strategy for airborne viruses like SARS-CoV-2 that enter through the respiratory tract.

IgY have high target specificity and binding avidity, making them remarkable at neutralizing pathogen activity in the respiratory tract and lungs [6]. IgY do not activate antibody-mediated responses in humans and do not rely on host immune response for prophylaxis [6], thus protecting the most vulnerable patients, including immunocompromised, elderly, and young children.

Egg-derived IgY antibodies can be produced on a large scale and can be readily purified from egg yolk three weeks after the first hen immunization. Each immunized hen produces approximately 35 g of IgY per year; each immunized hen lays one egg per day for 8-10 months, with each egg containing ~ 100 mg of protective IgY, sufficient for multiple doses in humans [7]. The purified IgY can then be formulated into intranasal drops or nasal spray to block viral binding at the main site of entry, the nasal epithelium [8]. Although the use of IgY provides only transient protection and dosing must be repeated when individuals are at risk of exposure to the virus, it is effective immediately; unlike a vaccine, it does not require 5-6 weeks to mount protection. Treatment with IgY provides an immediate benefit, albeit for only a few hours, based on clearance time in the nasal mucosa [9,10].

Recent studies have explored the use of IgY to prevent SARS-CoV-2 infection by blocking and neutralizing the virus at its point of entry [11,12]. These studies suggest incorporating IgY into an intranasal or oral formulation could be an effective prophylaxis against SARS-CoV-2 [13]. However, although IgY may provide a safe and fast prophylactic approach, further development is needed so that it will be produced inexpensively and locally, at the origin of an epidemic.

IgY can be purified from egg yolks using methods involving water extraction and chemically-induced precipitation [6]. The existing IgY production methods require the use of relatively expensive equipment and non-common chemicals, and thus are a challenge for low-resource communities [14]. The aim of our study was to create an alternative IgY extraction method that is inexpensive and accessible to users without access to commercial laboratories and specialized reagents. By using low-cost and readily available materials, we set out to develop an open-source protocol that can be easily conducted anywhere in the world. This protocol and an accompanying suggested low-cost kit can enable the production of a means to stop viral spread, even in villages and local farms of low-to-middle-income countries. In the future, rapid, local IgY prophylaxis may be an optimal first-line response before a newly discovered virus can cause a global pandemic, or as an ongoing prophylactic strategy in resource-constrained settings.

## METHODS

### IgY extraction

**Figure 1:**
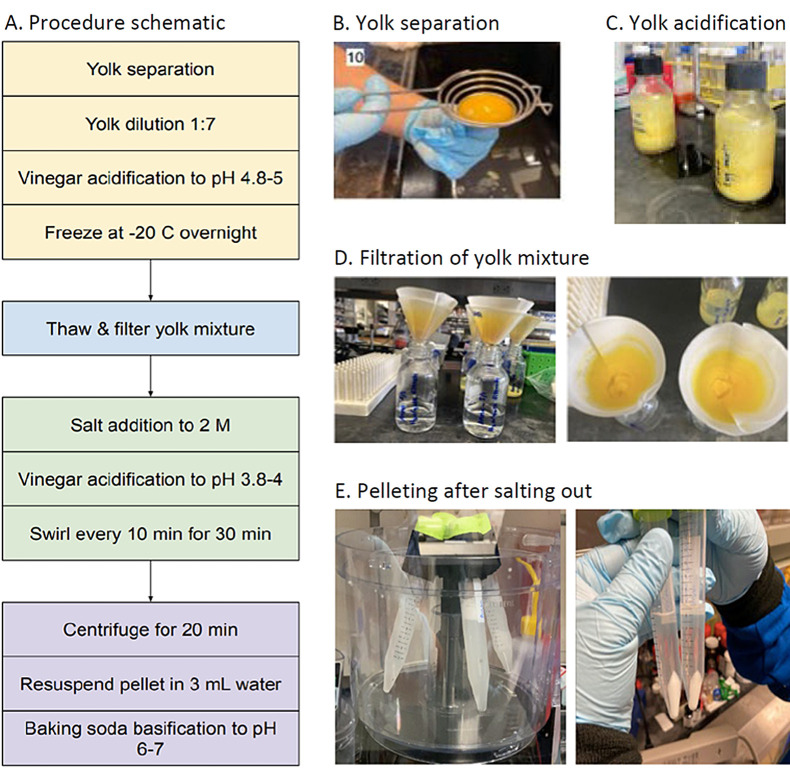
Summary of the IgY purification procedure. **Panel A.** Summary of steps in the IgY purification process. **Panel B.** Image of yolk separation from albumen, using an egg separator; leftover albumen are pulled off from under the separator using gloved hands. **Panel C.** 125 mL bottle filled with final solution from B. **Panel D.** Image of removal of yolk aggregates by filtration, after acidification and freezing of the mixture; yellow residues remained in the Whatman filter paper and the filtered solution in the bottle was clear. **Panel E.** Image of the makeshift centrifuge (left) and the final precipitated pellet in the 15 mL tubes.

We developed a detailed, stepwise protocol for IgY extraction from eggs of immunized hens using common household reagents and utensils. The IgY extraction process comprises five steps (**Figure 1****,** Panel A): yolk preparation and first acidification (1), filtration (2), salinification and second acidification (3), centrifugation (4), and resuspension and filling disposable receptacles for human use (eg, nasal dropper; 5). Sterile test-tubes, glass bottles, and single use nasal droppers were used. All glass and metal devices were sterilized by submerging in boiling water for at least 10 minutes. Water for yolk solution preparation should be boiled, cooled and stored in clean pre-boiled bottles until use.

#### Egg preparation and yolk extraction

We collected store-bought egg(s) from storage (refrigerator; >7°C), cleaned the shell of solids and dirt, and wiped with disinfecting wipes (Clorox® Wipes, Clorox Company, Oakland, CA, USA) or water diluted liquid disinfectant (Clorox Cleaner, 0.3% alkyl dimethyl benzyl ammonium chloride, Clorox Company, Oakland, CA, USA) at a ratio of 1 tablespoon liquid disinfectant per 2 cups water. A funnel was placed on top of a 15 mL sterile tube (8FYE3; W. W. Grainger, Inc., Lake Forest, IL, USA). After cracking the eggs, a yolk separator (Kitchen Stainless Steel Egg Yolk Separator Divider, 22.7 cm × 6.7 cm × 3 cm, Item 21001434, AliExpress, Hangzhou, China) and gloved hands were used to gently separate the yolk from the albumen without breaking the yolk sac (**Figure 1**, Panel B and Figure S1 in the **Online Supplementary Document**). The remaining albumen was gently pulled off from the yolk and discarded. The yolk separator containing the yolk was placed over the prepared funnel. A sharp object (eg, an individually wrapped toothpick; Royal Plain Individually Wrapped Toothpicks 1000 Ct) was used to gently break the yolk sac from the bottom, so that the yolk contents could drip through the funnel into the 15 mL tube. Once the liquid yolk contents were drained, the remaining yolk sac was discarded. The volume of the yolk content was then recorded using the 15 mL tube.

#### Yolk dilution, acidification and filtration

The yolk was transferred to a 125 mL glass bottle (Avantor/VWR® Media/Storage Bottles with GL Screw Caps, Radnor, PA) and diluted with room temperature, pre-boiled tap water using a 7:1 water to yolk ratio; approximately 85-100 mL water was added. The pH of the yolk mixture was adjusted to 4.8 by adding white vinegar (Better Living Brands, Pleasanton, CA) dropwise. The bottle was capped and gently shaken for ~ 15 seconds. Using pH 0-6 strips P4661; (Sigma-Aldrich, Inc.; St. Louis, MO), the pH of the solution was determined and further adjusted by adding one drop at a time of vinegar to the solution and retesting the pH after each addition (**Figure 1**, Panel C). The bottled yolk solution was then placed at -20°C until the solution froze solid (about 2 hours).

The frozen yolk-containing bottle was placed in a room temperature water bath. After it thawed completely (approximately 2 hours), the solution was filtered through folded Whatman paper (150 mm diameter, cat. number: 1002-150, lot number: 17083520, Millieporesigma; **Figure 1**, Panel D and Figure S2 in the **Online Supplementary Document**) placed on a funnel in the opening of a clean 125 mL glass bottle to obtain visually clear filtrate. If the filtrate was yellowish, the entire procedure was repeated.

#### Salinification

The filtrate volume was measured using the volume marks on the glass bottle and the number multiplied by 0.12 to determine the amount of table salt (NaCl; in mg dry weight) to be added. The average amount of salt per single-serve iodized salt packet (N’JOY Iodized Salt Packets 0.5g, N’JOY) was around 0.6 g, and our filtrate volume was around 80-90 mL. We therefore used about 20 salt packets ( ~ 11-12 g of salt) per trial to reach the desired 2M NaCl in the filtrate. The solution was mixed gently until the salt fully dissolved. Using pH paper strips, the solution pH was then adjusted to 3.8-4 using white vinegar, added dropwise. The solution was shaken gently for ~ 15 seconds, pH adjusted as needed, and the solution was gently swirled every 10 minutes for half an hour.

#### Centrifugation

Four 15 mL centrifuge tubes (8FYE3; W. W. Grainger, Inc., Lake Forest, IL, USA) were filled with equal volume of the solution above and centrifuged using a Beckman Coulter centrifuge (Avanti J-26 XP, Indianapolis, IN) at 1300 g for 10 minutes.

While there have been other ultra-affordable centrifuges described previously, they either cannot accommodate the large volume needed for our application [15], or require a significant amount of technical skill and assembly [16]. We therefore developed an improvised, tabletop centrifugation device engineered from a low-cost, mass-produced food processor (Hamilton Beach 70740 8-Cup Food Processor, Hamilton Beach Brands, Glen Allen, VA, USA; **Figure 1**, Panel E and Figure S3A in the **Online Supplementary Document**). An adapter to hold the centrifuge tubes was designed using cloud-based computer-aided design software and printed on a 3D printer. The alternative centrifugation device comprising the food processor and adapter is shown in Figure S3 in the **Online Supplementary Document**, and its design and prototyping is described in Supplemental Methods.

The adapter was mounted onto the food processor, which was set to speed 1 and turned on for 5-15 minutes, until the pellet size no longer increased (usually 5 minutes; **Figure 1**, Panel E). After centrifugation, the supernatant was gently decanted and each pellet was resuspended in 3 mL previously boiled, cold water by gently pipetting up and down without foaming.

#### Neutralization of IgY solution and filling a dispenser with IgY for human use

The pH of the solution was increased to 6.5-7.5 by slowly adding dry baking soda (Arm and Hammer Inc.; York PA) grainwise. pH was confirmed using pH 4-7 strips. 2-3 mL single use nasal droppers (eg, Item #: 66437, United States Plastic Cor; Lima, OH) can then be filled with about 1.5 mL solution and capped tightly. These single day-use droppers can be kept at room temperature for up to 2 weeks and for at least 6 months at 4°C, without changing the quality of the purified IgY.

### Experimental verification of IgY extraction and purification

#### Gel electrophoresis and Western blot analyses

Stain-free 4%-20% SDS PAGE (Criterion TGX Stain-Free Protein Gel; #5678094; BioRad, Hercules, CA) and Western Blot analysis were used to characterize the purified IgY. The two primary antibodies used were: rat anti-IgY (Sapphire, LO-IgY-16, batch: 10332, Ann Arbor, Michigan), diluted 1:500 and mouse anti-ovalbumin (Abcam; ab17293, lot number: GR3376118; Cambridge, MA) diluted 1:500, followed by goat anti-rat HRP (EMD Millipore; AP136P, Lot. Number: 3660343; Billerica MA) Cat. No: NA931V (Rock Immunochemicals; RL610603002, Lot. No: 17041904; Pottstown, PA) and sheep anti-mouse HRP, respectively.

## RESULTS

To create an easy, fast method that allows antiviral IgY production in any location, with the goal of producing a low-cost prophylaxis, we modified existing protocols of IgY purification from eggs [14], summarized in **Figure 1**, using common household reagents including table salt, vinegar and baking soda (see Table S1 in the **Online Supplementary Document** for replacement items from standard protocols). We also re-engineered a prototype centrifuge, made of a food processor and a 3D printed plastic attachment, to hold the tubes during centrifugation (**Figure 1**, Panel E and Figure S3 in the **Online Supplementary Document**).

**Figure 2:**
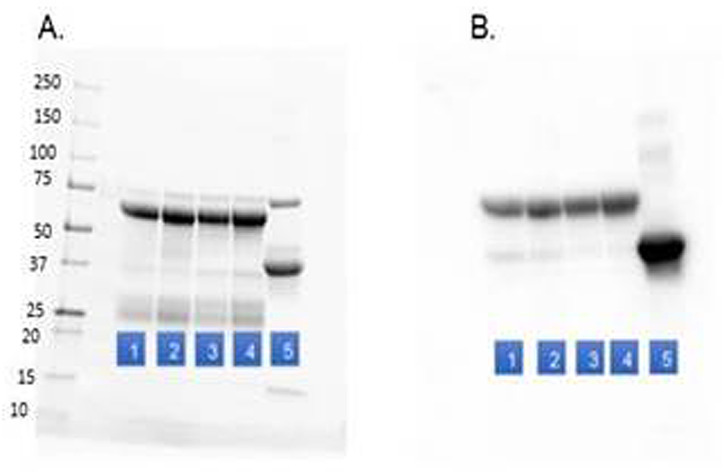
Experimental confirmation of IgY purification. **Panel A.** Stain-free SDS-PAGE of lane 1-4: preparations of purified IgY using the new protocol. Lane 5; purified ovalbumin from the albumen. Each at 0.5 ug protein/lane. The upper and lower proteins in lanes 1-4 are the heavy and the light chains of IgY, respectively. **Panel B.** Western Blot analysis with anti-IgY and anti-ovalbumin identifying the heavy chain of IgY in lanes 1-4 (upper band) and ovalbumin (lower band), in lane 5. The levels of ovalbumin in the IgY preparations (lanes 1-4) were almost undetected (compared to lane 5; purified albumin) especially in the later preparations (lanes 3,4).

The IgY preparation protocol including the improvised centrifuge provided a high yield and highly pure IgY. Characterization of the purified IgY via SDS-PAGE and Western Blot is shown in **Figure 2** and IgY yield was calculated based on protein determination. Egg yolks of commercial hens contain about 100 mg IgY [6], and the calculated average yield of purified IgY per egg using the modified protocol and improvised centrifuge was 90 ± 15 mg (about 90% yield), based on six independent preparations conducted by three operators. (Four of the six preparations are shown in **Figure 2**.)

A benchtop centrifuge should be used for IgY purification if available. However, centrifuges are expensive (US$1000 - US$5000), their shipment is costly due to their size, and they may not be available in more rural areas where epidemics or pandemics remain a threat. We therefore used a low-cost small food processor fitted with a 3D printed adapter to generate a centrifuge powerful enough to purify antibodies (Figure S3 in the **Online Supplementary Document**). The final adapter design of the improvised centrifuge could withstand multiple centrifugations at intervals ranging from 5 minutes to 20 minutes. Each precipitation round used a total of 50-60 mL of IgY solution in four 15 mL test tubes and resulted in easily separated pellets (**Figure 1**, Panel E).

To evaluate the efficiency of our setup, a tachometer (Digital Tachometer DT-2234C+, AGPtek, Brooklyn, NY, USA) was used to measure an rpm of 4213 for the processor. With a radius (r) of 6.6 cm from the axis of rotation to the edge of the centrifuge tube, the maximum g force (RCF) of our centrifugation system is estimated to be 1382 (Equation 1). This is comparable to the force recommended by the published procedure [14].

*RCF = RPM^2^ × (1.118 × 10^−5^) × r_cm_* (Eq. 1)

The limitations of this solution are discussed below.

### Design and cost of an IgY purification kit

**Figure 3:**
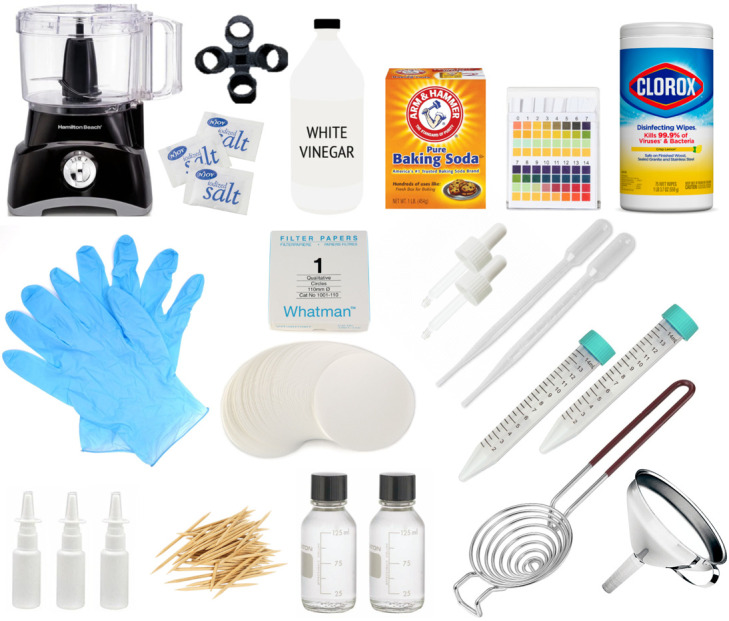
Components of the IgY kit. All kit materials listed in **Table 1** are shown not to scale. All kit materials except the food processor easily fit into a small (15 × 15 × 15 inch) box.

We also designed a kit for IgY preparation and a step-by-step protocol for its use (Supplementary Materials). Each kit will contain a complete supply of materials to purify IgY from 100 eggs. Kit contents are listed in **Table 1** and an image of the complete contents is shown in **Figure 3****.**

**Table 1:** Kit materials and cost estimate

Material	Disposable?	Price/Bulk	Price/Unit
**Preparation of immunogen**	Yes	(1L × US$200)/(100 × 30)	US$0.06
**Hen and housing:**			
Hen	No	US$3.76	US$3.76
Monthly cost of hen	No	US$4.50	US$4.50
**Kit:**			
White vinegar (4 drops)	Yes	US$0.02 (US$0.02/fl oz)	US$0.02
Boiled water	Yes		
Table salt (sodium chloride) (11g)	Yes	US$0.02	US$0.02
Pipette × 2	Yes	US$0.04 × 2 = US$0.08	US$0.08
Disinfectant wipes (Chlorox)	Yes	US$0.08	US$0.08
Whatman filter paper (150 mm diameter)	Yes	US$0.07	US$0.07
Metal funnel	No	US$6.90	US$6.90
pH strips	Yes	(US$0.14 × ~ 6) = US$0.84	US$0.84
Toothpick	Yes	US$0.00	US$0.00
Baking soda (1/1000)	Yes	US$0.58	US$0.00
Egg separator	No	US$2.49	US$2.49
15 mL centrifuge tube (disposable)	Yes	US$0.25 × 4 = US$1.00	US$1.00
125 mL glass bottle	No	US$2.85 × 2 = US$5.7	US$5.70
Nose droppers	Yes	US$0.62 × 2	US$1.24
Food processor (Hamilton Beach, Model No. 70740)	No	US$44.99	US$44.99
3D printed attachment (57grams)	Yes	US$1.10	US$1.10
Ender 3 3D printer	No	US$189.00	US$189.00
Nasal sprayer	Yes	US$1	US$1.00
Gloves (2 per prep)	Yes	US$0.1699 × 2 = US$0.33	US$0.33
Total kit (disposable)	US$5.84		
TOTAL KIT × 100 eggs	US$584.36		
**Cost kit per dose (/2500)**	**US$0.23**		
COST of 100 HENS + HOUSING	US$826.00		
Total kit + non-disposable + hens	US$1656.95		
**Total cost per dose (/2500)**	**US$0.66**		

We calculated a cost estimate (shown in **Table 1**) of the kit containing enough disposables to purify IgY from 100 eggs, which is equivalent to about 2500 doses, assuming 25 doses per egg or 5 daily doses/person. This calculation assumes each dose contains 2 mg IgY, based on a 0.1 mL dose of 20mg/mL solution per nare, multiplied by two. We also assume that each person will need to redose up to 5 times per day when at risk of exposure.

Where needed, an adapter for the food processor can either be produced at a local facility with a 3D printer, such as a central university prototyping laboratory near the distribution location, or will be provided with items in the kit. The 3D printer used in this study has a current list price of US$179, making it relatively affordable to acquire by the local producer even if for this specific application.

**Table 1** provides a cost estimate of 100 hens, each receiving two immunizations in a hen growing facility, to produce a total of 100 eggs/d or 25 000 doses/d, and the cost of immunogen production (2 × 50 µg of immunogen per hen [12]).

For comparison, an estimate of the cost to produce IgY from 100 eggs using Specific Pathogen Free (SPF) chickens at a commercial clinical facility is shown in **Table 2****.** We had previously investigated IgY production using SPF hens at a contract research organization (CRO) facility. The expense of such IgY production led us to develop an IgY purification kit to reduce costs. Based on costs and estimates provided by our CRO partners for IgY production under Good Manufacturing Practices (GMP), we calculated the cost of each dose is US$5.40, compared with a cost per dose of US$0.66 using our kit.

**Table 2:** Cost estimate of IgY production at a commercial facility

Material	Total price	Price/100 eggs
Preparation of protein	(1L × US$200)/(100 × 30)	US$100.00
Hen and housing & IgY extraction	1 hen at US$28 000 for 8 mo	US$11 666.66
Cost of formulation and fill	840 eggs at US$10 000	US$1190.48
Batch release test	840 eggs at US$1000	US$119.05
Vials	1600 vials at US$1000 (3.75 doses per vial)	US$416.69
TOTAL COST per 100 eggs	US$13 492.88	
**Cost kit per dose (/2500)**	**US$5.40**	

## DISCUSSION

We developed an easy and fast step-by-step method to produce IgY antibodies from egg-laying hens as a low-cost prophylaxis to combat viral epidemics early and help combat ongoing pandemics.

The primary aim of this project was to modify previous IgY purification protocols by providing substitutions for expensive, hazardous or otherwise inaccessible materials and equipment for use in resource-limited regions. Using common household and safe ingredients (eg, replacing 0.5N HCl with salad vinegar), we devised a step-by-step protocol that can make IgY production accessible to users with no access to commercial biolaboratories. Our protocol to purify IgY from egg yolks provided a high yield and a highly enriched IgY preparation. Together, this method constitutes three important improvements over other prophylaxes. First, the cost of IgY prophylaxis relative to other prophylaxes like vaccines is much lower. Second, by enabling local production, the prophylaxis no longer depends on the use of unreliable and expensive distribution systems. Lastly, the use of household reagents and providing a prepared kit will make IgY prophylaxis even more accessible relative to IgY production and purification in a commercial facility. The protocol that we have provided can be replicated in a low-resource environment using commonly available, low-cost chemicals. Purified IgY from eggs of hens immunized with proteins from a known pathogen, such as SARS-CoV-2, can then be readily formulated as an intranasal prophylactic to slow disease transmission until active vaccines become available.

We suggest the use of immunized egg-laying hens as a source of antibodies against the infectious agent (eg, SARS CoV-2). Egg-laying hens are available throughout the world, are inexpensive and often are immunized against poultry-specific pathogens, such as Eimeria [17]. If the hens are immunized with recombinant proteins and not virus, the hens and their eggs are safe and non-infectious, and therefore can be handled by non-specialized growers. The eggs containing the protective IgY antibodies can be easily processed into a stable and easy-to-store lyophilized or refrigerated product locally, rather than in a specialized facility. Importantly, we show that IgY as a passive vaccine can be produced within a few hours, even in a rural facility and at a very low cost.

**Figure 4:**
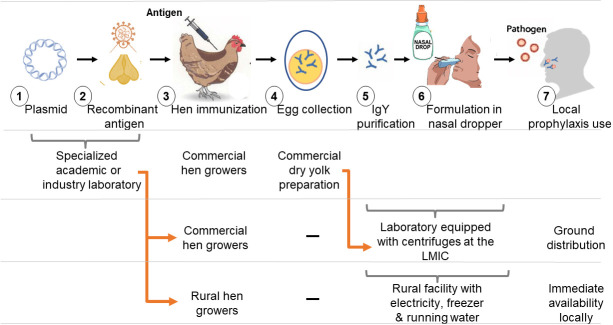
High-level implementation of the new IgY protocol and required facilities. A scheme illustrating the implementation of the IgY purification process in two LMIC settings: Laboratory equipped with centrifuges; and a rural facility with electricity, a freezer and running water. The chosen recombinant protein (immunogen) can be produced in a specialized academic or industry laboratory and shipped for immunization as dry material (highly stable and requires no special refrigeration). Hens can then be centrally immunized by a vet, or by rural hen growers, and the resultant IgY-containing eggs can be purified either in a central facility or at a rural setting. The centrifugation step can be performed either at the central laboratory facility, equipped with a centrifuge, or locally, using our repurposed centrifugation device and printed adapter. Finally, the purified IgY is formulated into dropper bottles and can be used intranasally.

How can we make use of this procedure? In the simplest form, should a country identify a threat of an epidemic or if a pandemic is already occurring, an antigen suitable for hen immunization should be produced in vitro or in *E coli* (**Figure 4**, steps 1 and 2) and sent with simple oil emulsion, such as paraffin oil used in Freund’s adjuvant [18] to local commercial chicken farms for immunization (**Figure 4**, step 3). Each egg-laying hen should be immunized twice, using a simple oil emulsion in a standard protocol [19] and eggs should be collected daily, starting 3 weeks after the first immunization. Egg shells should be cleaned and yolks separated and dried in a central site or directly processed (**Figure 4**, steps 4-6) as described in our protocol, using either a standard tabletop centrifuge with at least 1000 × g, or using the makeshift centrifugation device. Purified IgY should be formulated and used locally or shipped within the country and used every ~ 4 hours (**Figure 4**, step 7), while awaiting local availability of active vaccines. Note that dry egg yolk preparation is commonly made by the food industry and is stable for months, and the purified IgY solutions are stable at room temperature for at least one week and at 4°C for many months [6]. Due to travel limitations, we could not perform this protocol in the field. However, most of the work was carried out in the kitchens of the undergraduate students and only the confirmation studies were repeated in a standard laboratory.

We see two potential users of our protocol: First, an established laboratory, equipped with centrifuges, freezers and autoclaves. The second, a rural facility with electricity and a -20°C freezer. Because our protocol uses low-cost and safe materials that can be easily obtained outside the laboratory setting, our protocol can also be used in low-resource settings, where communities can reproduce the purification steps without the need for high-cost, pharmaceutical-grade components found mainly in laboratory settings. We replaced all laboratory-standard chemicals with ingredients that are also used for cooking; we used pH papers instead of a more expensive pH probe and we replaced an expensive centrifuge with a food processor fitted with an adapter. The kit that we designed will contain all of these elements required for purification, including hardware and disposables (**Figure 3**). Note that the cost estimate for the kit and final product in **Table 1** are based on US prices and without volume discount; we expect the final cost to be reduced up to a factor of 10, or about 6 cents/dose.

There are several limitations to our engineered centrifuge. When using the centrifuge, the lid of the food processor has to fit back on the safety mechanism in the central spindle space to operate. However, our current design does not provide this safety feature and during our testing phase, we circumvented the safety mechanism and conducted the centrifugation behind a shield, at least 15 feet away from the operator while in operation. This safety feature is insufficient and needs to be improved before the kit is sent to a facility with no access to a centrifuge.

The 3D-printed adapter has additional limitations: while up to three copies of the adapter can be printed simultaneously, the print time is lengthy ( ~ 7 hours). Issues with previous design iterations, such as low infills and erosion of the tube plastic against the side of the processor, were solved in design and 3D printing software in which the design was modified to decrease the angle of the 15 mL tubes and increase the print stability and durability. In addition, the shape of the spindle base on which the adapter sits is unique to Hamilton Beach processors and may not fit other food processors. We hope that food processor manufacturers could provide a safer solution and a metal adapter on which the test tubes can be mounted.

There are also concerns regarding sterility of the final product to be used as nose drops. Eggs should be properly cleaned to avoid contamination of the intranasal prophylaxis by scrubbing off dirt and wiping with Clorox Wipes or diluted Clorox solution, and all non-disposable equipment should be sterilized in boiling water. Our study is also limited in that our experiments have not confirmed the absence of viruses and pathogens in the egg of the commercial hens. Future studies should address this directly.

We also made the assumption that the site has access to electricity, clean water, and refrigeration. While our novel food processor centrifuge is significantly lower in cost than a traditional centrifuge, electricity is still necessary to power it. Several alternatives were explored, but none achieved a high enough rotational speed to simulate a centrifuge. Clean water and refrigeration are also needed to dilute the yolk and store the IgY, respectively. Unfortunately, there are still many low-resource communities that do not have reliable access to these. Finally, our protocol has not been fully tested by a third party to ensure that it is truly reproducible.

The intranasal safety of reagents used in similar protocols demonstrate the safety of IgY, and since common household ingredients are used in low amounts, the final product is unlikely to cause any nasal irritation. However, safety to the nasal mucosa needs to be determined in proper clinical studies. We also need to consider whether a more viscous formulation is needed for optimal effect of the nasal drops and whether the antiviral IgY is efficacious as prophylaxis. A human safety study of intranasal IgY prophylaxis against SARS-CoV-2 of up to 2mg/IgY/nare three times daily for 14 days has been completed (registered at clinicaltrials.gov; NCT04567810). If proven safe and effective, this protocol may provide a blueprint for production of other airborne antiviral agents.

## CONCLUSION

Our study shows that chicken-derived IgY antibodies can be quickly and easily extracted and purified from egg yolks of immunized hens using common household reagents and tools. We outlined detailed, step-by-step methods for a user with no access to a commercial facility to produce protective IgY. Our protocol is the first step to producing IgY as an anti-airborne virus prophylaxis in resource-limited geographical areas as a means to respond to new infectious viruses or reduce viral transmission during epidemics/pandemics, before vaccines are available. Its utility can be first tested as a temporary solution for the continued spread of SARS-CoV-2 globally. Finally, the procedure outlined here can readily be modified to fight against other future viral pandemics and its application warranted further research. Future plans include identifying a partner in a resource-constrained setting to collaborate on a pilot to further validate the protocol and assess the viability of our approach.

## Additional material


Online Supplementary Document
