# Supplementary material for: Affordable IgY-based antiviral prophylaxis for resource-limited settings to address epidemic and pandemic risks
Source: J Glob Health. 2022 Feb 26;12:05009. doi: 10.7189/jogh.12.05009 (PMC8877785; doi:10.7189/jogh.12.05009)
Supplement: Online Supplementary Document [file jogh-12-05009-s001.pdf]

SUPPLEMENTAL MATERIALS

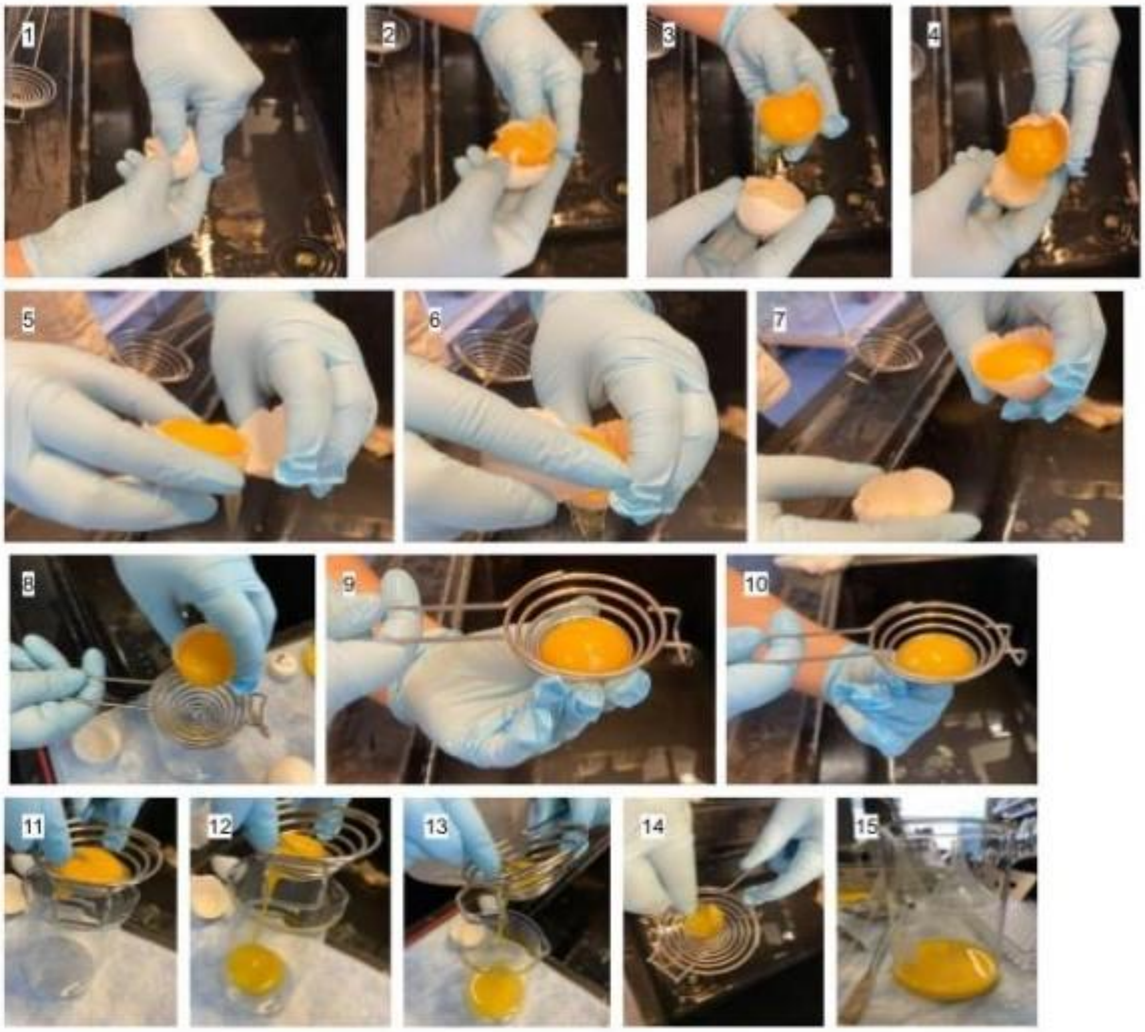

Figure S1. Early steps of the egg yolk preparation process: cracking the egg (1-3), alternating between egg shells to separate yolk and albumen (4-7), separating with an egg separator (8-11), puncturing egg yolk membrane and draining (11-15).

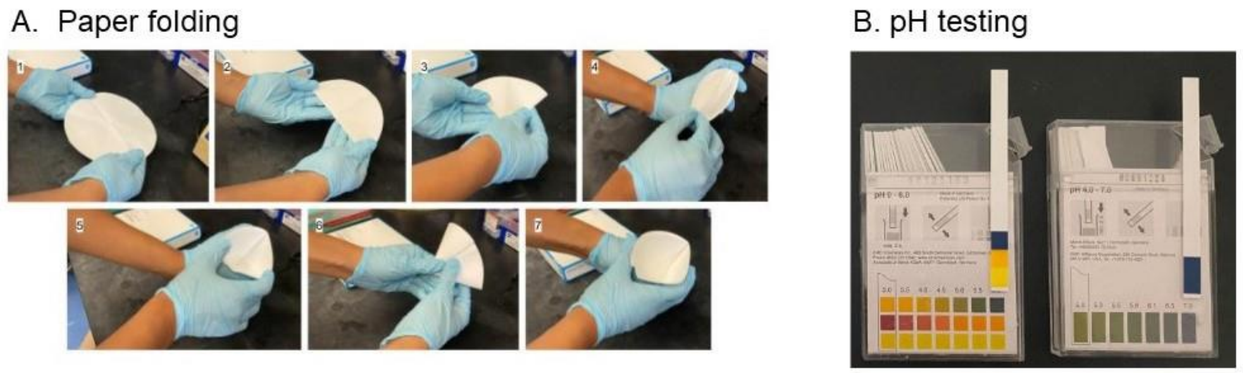

Figure S2. A) Filtration of the yolk aggregates through a folded Whatman filter paper. Images show how to fold the paper into a cone so it fits over the top of the bottle. To create the cone, the paper is folded into fourths, and an opening is created by pulling the paper apart and pressing half of the paper to the outside of the other half to shape into a cone. **B)** pH strips are used to determine the pH of the IgY mixture. Where available, a pH meter should be used instead.

Supplemental Table S1: Comparison between standard preparation and the modified preparation developed here.

Step	Previous Work	Modified Protocol
Acidification #1 to pH = 4.8 - 5.0	0.5M HCl	5-8% acetic acid (Vinegar)
Acidification #2 to pH = 3.8 - 4.0	0.5M HCl	5-8% acetic acid (Vinegar)
Basification to pH = 7.5	PBS	Baking soda added as solid

Supplemental Methods:

Step by Step Instructions to be provided with the kit:

BEFORE YOU BEGIN

1. Wash all provided glass and metal supplies with water and diluted soap before each use.
2. Boil water and immerse all provided glass and metal supplies, including the metal funnel, egg separator, and glass bottles.
3. After 15 minutes of boiling, cover the pot and let water cool.
4. Remove one glass bottle filled with clean boiled water and cap.
5. Remove the rest of the clean supply and place on disposable clean paper until use.

YOLK EXTRACTION (Supplemental Figure S1):

1. Collect egg(s) from storage (cold room or refrigerator) and wipe with diluted Clorox® or a similar cleaning product.
2. Collect 1 funnel, 1 egg yolk separator, 1 15 mL tube, and 1 toothpick. Place the funnel on top of the 15 mL tube.
3. Crack the egg and separate the yolk from albumen (egg white). Use the yolk separator or your hands to gently remove remaining albumen from yolk without breaking the yolk sac.

   Recommended Strategy: Use fingers to gently grasp egg white and pull from yolk, pass yolk between egg shells, or pass yolk between hands and scoop away remaining egg white (Figure 1B and Supplemental Figure S1).
4. Place the separated yolk on the yolk separator. Place the yolk separator over the funnel. Use a sharp object (e.g., disposable toothpick) to gently break the yolk sac from the bottom and allow yolk contents to drip through the funnel into a 15 mL tube. Once liquid yolk contents have been drained, discard the remaining yolk sac (Supplemental Figure S1).
5. Record volume of yolk content using the 15 mL tube marks.

FIRST ACIDIFICATION:

1. Dilute yolk with room temperature, pre-boiled tap water using a 7:1 water to yolk ratio.
   a. Drain yolk contents from 15 mL tube into the 125 mL bottle.

b. Add 7 volumes of cold (previously boiled) water using the 15 mL tube as the measuring vessel; about 85 mL. When finished, all egg yolk and water should fit in the 125 mL bottle and the 15 mL tube should be empty (Figure 1C).

2. Adjust the pH of the solution to 4.8 using white vinegar.
   a. Use a disposable glass pipette to add 3 drops of vinegar to the solution.
   b. Cap the bottle and swirl the solution gently for ~15 seconds.
   c. Use the pH 0-6 strips to test the pH. If the pH is above 4.8, repeat steps 2 a-c, adding 1 drop at a time (Supplemental Figure S2B).
3. Freeze the solution in 125 ml bottle at -20°C until solid (~2hrs, time variable based on the freezer).

FILTRATION:

1. Remove 125 mL bottles from the freezer and place bottles in a bowl filled with room temperature water. Make sure that the bottle does not float and the cap is not impressed in the water. Wait for the solution to thaw completely (~2 hours).
2. Filter the solution: place a funnel in the opening of the new 125 mL glass bottle. Fold the filter paper into quarters and pull open one outer edge to form a cone. Place the filter paper cone into the funnel. Pour the solution into the filter paper and wait for it to filter through (Supplemental Figure S2A).
3. Ensure that filtrate in the bottle is clear (Figure 1D).

SALINIFICATION AND SECOND ACIDIFICATION:

1. Salinification
   a. Measure the volume of the solution. Multiply the volume by 0.12 to determine the amount of salt added using the formula: Volume solution x 0.12 = grams of salt. (The kit will indicate the weight of each salt package.)
   b. Add the calculated amount of salt. Gently swirl the solution to mix until salt has fully dissolved.
2. Adjust the pH of the solution to 3.8-4.0 by adding white vinegar dropwise.
   a. Use a transfer pipette to add 2 drops of vinegar to the solution.
   b. Shake the solution gently for ~15 seconds.

c. Test the pH with the pH 0-6 strips. If the pH is above 4, repeat steps a-c (Supplemental Figure S2B).

3. Swirl the solution gently every 10 minutes for half an hour.

CENTRIFUGATION

1. Collect four 15 mL centrifuge tubes.
2. Divide the volume of the total solution in the glass bottle by 4 and pour that amount of the solution into each centrifuge tube. Confirm that an even amount of liquid is in each of the four tubes.
3. Mount the tube adapter to the centrifuge (Figure 2).
4. Load the centrifuge tubes into the centrifuge.
5. Lock the lead and secure the centrifuge to the table.
6. Set centrifuge to speed 1 setting.
7. Turn on the centrifuge using a power strip.
8. Spin for 5-15 minutes, until pellet size does not increase further.
9. After centrifugation, gently pour out supernatant and avoid disturbing pellet (Figure 1E).
10. Resuspend centrifuged pellet in 3 mL water: Take up 3 mL of water with a new pipette. Take up and release the water into the pellet as many times as necessary until the pellet is suspended in water and no longer solid.
11. Gradually add baking soda grainwise to raise the pH of the pellet to 6-7 and test using the pH 4-7 strips.

This material provides about 12 mL of purified IgY – enough for 5-6 individuals for 4 doses (every 4 hours/day), assuming 20mg/mL of IgY at 0.1 mL drop/nare.

FILL INTO SINGLE DAY DROP DISPENSER

1. Use the 2 mL disposable pipettes to fill the disposable nose dropper halfway.
2. Each drop bottle has enough material for one user for one day.
